# Measurement and Calibration of Plant-Height from Fixed-Wing UAV Images

**DOI:** 10.3390/s18124092

**Published:** 2018-11-22

**Authors:** Xiongzhe Han, J. Alex Thomasson, G. Cody Bagnall, N. Ace Pugh, David W. Horne, William L. Rooney, Jinha Jung, Anjin Chang, Lonesome Malambo, Sorin C. Popescu, Ian T. Gates, Dale A. Cope

**Affiliations:** 1 Department of Biological and Agricultural Engineering, Texas A&M University, College Station, TX 77843, USA; thomasson@tamu.edu (J.A.T.); gcbagnall@outlook.com (G.C.B.); 2 Department of Soil and Crop Sciences, Texas A&M University, College Station, TX 77843, USA; npugh@tamu.edu (N.A.P.); dwhorn3692@tamu.edu (D.W.H.); wlr@tamu.edu (W.L.R.); 3 School of Engineering and Computing Sciences, Texas A&M University-Corpus Christi, Corpus Christi, TX 78412, USA; jinha.jung@tamucc.edu (J.J.); anjin.chang@tamucc.edu (A.C.); 4 Department of Ecosystem Science & Management, Texas A&M University, College Station, TX 77843, USA; mmoonga@tamu.edu (L.M.); s-popescu@tamu.edu (S.C.P.); 5 Natural Resources Institute, Texas A&M University, College Station, TX 77843, USA; ian.gates@ag.tamu.edu; 6 Department of Mechanical Engineering, Texas A&M University, College Station, TX 77843, USA; drdcope@tamu.edu

**Keywords:** fixed-wing UAV, sorghum plant height, structure from motion, multi-level GCPs, GCP-based height calibration, image blurriness, wind speed

## Abstract

Continuing population growth will result in increasing global demand for food and fiber for the foreseeable future. During the growing season, variability in the height of crops provides important information on plant health, growth, and response to environmental effects. This paper indicates the feasibility of using structure from motion (SfM) on images collected from 120 m above ground level (AGL) with a fixed-wing unmanned aerial vehicle (UAV) to estimate sorghum plant height with reasonable accuracy on a relatively large farm field. Correlations between UAV-based estimates and ground truth were strong on all dates (R^2^ > 0.80) but are clearly better on some dates than others. Furthermore, a new method for improving UAV-based plant height estimates with multi-level ground control points (GCPs) was found to lower the root mean square error (RMSE) by about 20%. These results indicate that GCP-based height calibration has a potential for future application where accuracy is particularly important. Lastly, the image blur appeared to have a significant impact on the accuracy of plant height estimation. A strong correlation (R^2^ = 0.85) was observed between image quality and plant height RMSE and the influence of wind was a challenge in obtaining high-quality plant height data. A strong relationship (R^2^ = 0.99) existed between wind speed and image blurriness.

## 1. Introduction

Continuing population growth will result in increasing global demand for food and fiber for the foreseeable future. In the near term, an increase of almost one-third or 2.3 billion people along with improving living standards are anticipated through midcentury [1]. Potential effects of climate change may also affect agricultural production especially in a regional context, which may require adjustments in farming practices and production technology [2]. Two critical research efforts have the potential to meet world agricultural productions such as crop improvement through plant breeding and genetics and production optimization through precision-agriculture management strategies [3,4]. In both cases, the measurement of numerous traits such as plant height, leaf-area cover, and crop density is essential for increasing yield potential and protection from crop losses.

Tremendous advances are being made in high-throughput plant phenotyping (HTPP) technology by enhancing the technologies available for crop improvement [5]. Ultimately, breeders and geneticists hope to use HTTP to increase the efficiency of phenotyping [6,7]. Some of these HTPP techniques are translatable to precision agriculture specifically plant height estimation with 3D point clouds generated from high-resolution imagery. In HTPP, sensors incorporating measurement techniques including visible or near-infrared reflectance or fluorescence can be carried on automated platforms to more efficiently estimate important traits in order to accelerate breeding and genetics research. Multiple types of imaging sensors such as RGB, multispectral, thermal, and Light Detection and Ranging (LIDAR) are now used with unmanned ground vehicles (UGVs) or unmanned aerial vehicles (UAVs or “drones”) for mapping phenotypes at the plot or plant level [8,9,10]. A large number of potential metrics including spectral reflectance, thermal emittance, and plant height demonstrate great potential for the use of UAVs in vegetation monitoring [11,12,13]. With UAVs equipped with multispectral or hyperspectral sensors, the data are often used to build a vegetation index [14,15,16,17,18], which is an indicator of plant vigor, canopy cover, leaf area index (LAI), disease incidence, plant nutrient levels, and even biomass yields. Biomass monitoring, which commonly involves pre-processing of multispectral images including radiometric correction, geometric correction, and image enhancement, is crucial to breeders for yield prediction in order to improve crops and to growers since it affects agricultural management practices [19,20,21]. Bendig et al. [22] used combined linear regression models to estimate biomass (R^2^ = 0.9) with RGB vegetation indices in the early growth stages of maize. Vegetation indices from these sensors can potentially be used for making decisions and performing actions in farm management [23,24]. Since they can collect multiple images over the same area during a flight, UAVs can also help determine plant height, which is useful in assessing the influence of environmental conditions on plant performance and is an important phenotype for crop improvement and production optimization. 

During the growing season, variability in the height canopy of crops provides important information on plant health, growth, and response to environmental effects. Recent studies have shown that crop height can be derived from 3D dense point cloud data derived from the structure from motion (SfM) [25,26]. High resolution images have been shown to improve the plant-height model accuracy [27,28]. Willkomm et al. [29] generated models with spatial resolutions of 0.5 cm and found that modelled plant heights were on average 10 to 20 cm shorter than ground truth estimates. Some reasons for height underestimation were determined to be negative heights of ground surfaces in the crop surface model (CSM) and depression of the plant canopy affected by wind was caused by the movement of UAV rotors. More recently, Malambo et al. [30] found a high correlation between the digital surface model (DSM) obtained through SfM with rotary-wing unmanned aerial systems (UAS) image data and terrestrial laser scanning (TLS) from a spray tractor for the purpose of detecting maize and sorghum plant height. Results highlighted the potential for reducing laborious manual height measurement through rotary-wing UAS and SfM. In order to develop agricultural utility, it is important to develop accurate plant-height measurement capability with fixed-wing aircraft, which can cover larger acreages per flight, can also fly higher and faster, and may require a smaller number of images to get adequate ground coverage.

While UAVs can be used to collect very high-resolution data with various sensors and the data can be used for 3D visualization on a large scale [31], their disadvantages include limitations associated with weather, flight time and area coverage, and official permission to fly [32,33]. Consumers tend to use rotary-wing UAVs but fixed-wing models are not uncommon in agricultural research because they obtain lift from their wing surfaces and can typically cover larger areas on a single battery charge. This fact also makes fixed-wing UAVs attractive for the potential use at larger production farm fields. However, they fly faster than rotary-wing UAVs, which adds difficulty to the selection of an appropriate consumer-grade sensor because higher sensitivity and faster response may be required. The quality of the UAV-based image can be degraded due to image motion blur whether rotary-wing or fixed-wing UAVs are used. Image motion blur caused by camera movement during image acquisition under strong winds and turbulence is a significant obstacle to automatic data processing based on UAV imagery [34]. Several procedures including registration, orthomosaic generation, and 3D point cloud generation by SfM may be significantly affected by the image motion blur [35]. One specific issue is that sufficient tie point correspondence for successful image matching is a critical factor for achieving high-quality 3D models in common software like PhotoScan or Pix4D mapper. Tie point matching can also be made difficult by uniformity of pixels in flat terrain [36].

The overall goals of this research are to develop methods that simplify UAV remote sensing for eventual use in production agriculture and to maximize the reliability of the data in both crop improvement and production optimization. The specific objectives of this study were (1) to evaluate sorghum plant height estimates with SfM from a fixed-wing UAV that can cover a relatively large research field in one flight, (2) to evaluate improvements in plant height accuracy with height calibration based on ground control points (GCPs) having multiple known height levels, and (3) to identify remaining sources of error in plant height estimates.

## 2. Materials and Methods

### 2.1. Trial Plots

#### 2.1.1. Experimental Setup

**Figure 1:**
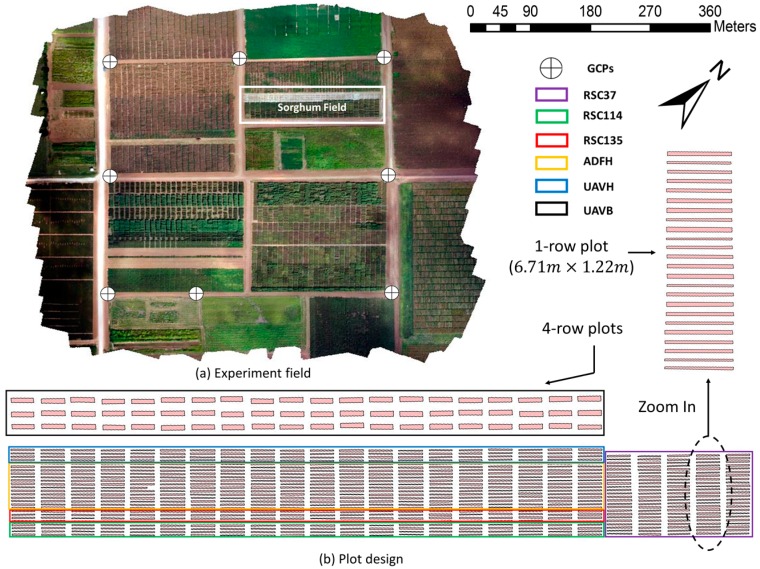
(**a**) Experiment field at Texas A&M AgriLife Research Farm. (**b**) Overview of the plot design in sorghum field with 700 plots.

A 180-m by 40-m sorghum field at Texas A&M AgriLife Research’s Brazos Bottom research farm (headquarters at latitude 30.549635 N, longitude 96.436821 W in WGS-84 coordinate system) near College Station, TX, USA (Figure 1a) was used for the plant height measurement experiment. The total size of the entire field area covered during the experimental fixed-wing UAV flights was 0.28 km^2^. The regional climate is categorized as temperate with an average annual temperature of 20.5 °C and average precipitation of 1018 mm. Six different types of sorghum germplasm were included in the experimental tests: one elite hybrid sorghum (ADFH), one historical elite hybrid (UAVH), three exotic early-program hybrids (RSC135, RSC37, and RSC114), and one bioenergy sorghum (UAVB). Each test was planted on April 1, 2017 and consisted of one-row plots measuring 6.71 m long with 1.22-m alleys except for UAVB, which consisted of four-row plots. The six tests had four replications each and altogether composed 700 plots (Figure 1b) in a randomized complete block design. Standard agronomic practices for grain sorghum and bioenergy sorghum in central Texas were employed.

#### 2.1.2. Ground-Truth Measurements of Plant Height

**Figure 2:**
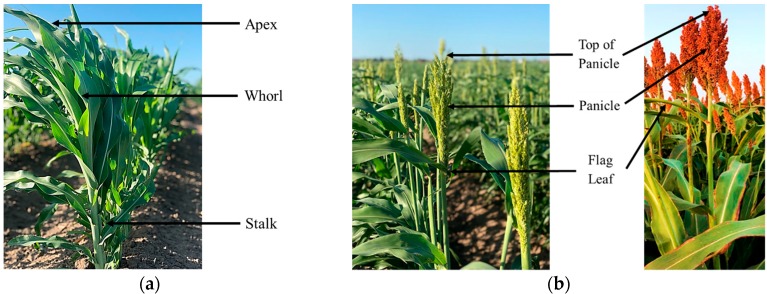
Ground truth measurement of plant height at two different growth stages (Photo Credit: Kayla Brock). (**a**) Vegetative stage. (**b**) Reproductive stage.

Ground-truth height (m) measurements were recorded manually with a meter stick. For plants that had not yet emerged from the early vegetative stage (whorl), measurements were taken from the ground vertically to the apex (highest point) of the plant (Figure 2a). For plants that had reached a reproductive stage, measurements were taken from the ground near the stalk and followed to the tip of the panicle (Figure 2b). Both measurements were essentially considered to be apex measurements. Therefore, they were treated the same relative to the UAS-derived measurements. Ground-truth measurements were recorded weekly or biweekly from May 26 to July 27 at the front of each plot (Table 1) by looking across the sorghum apices or panicles to get an estimated mean of the entire plot.

### 2.2. Image Data Acquisition

#### 2.2.1. UAV Platform

**Figure 3:**
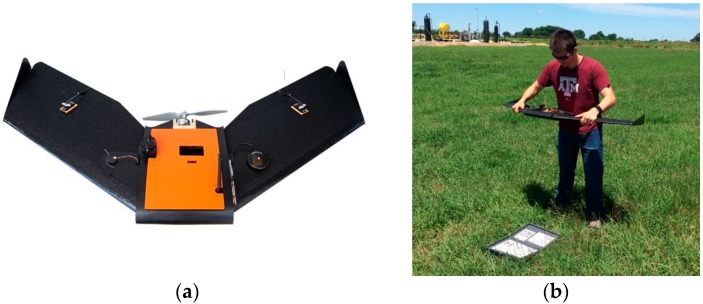
The fixed-wing UAV used in this study. (**a**) Tuffwing fixed-wing UAV. (**b**) The UAV in the take-off position.

The UAV used in this study was a Tuffwing Mapper (TuffWing LLC, Boerne, USA) fixed-wing UAV, which is a ready-to-fly kit with semi-autonomous horizontal take-off and landing (HTOL) (Figure 3). The Tuffwing weighs 1.9 kg and has a wingspan of 1.22 m and a maximum endurance of 40 min (Table 2). This UAV can perform user-defined waypoint flights with a differential global navigation satellite system (GNSS) receiver. It uses a brushless DC propeller motor powered by a lithium polymer battery with a capacity of 6200 mAh.

#### 2.2.2. Sensor

**Figure 4:**
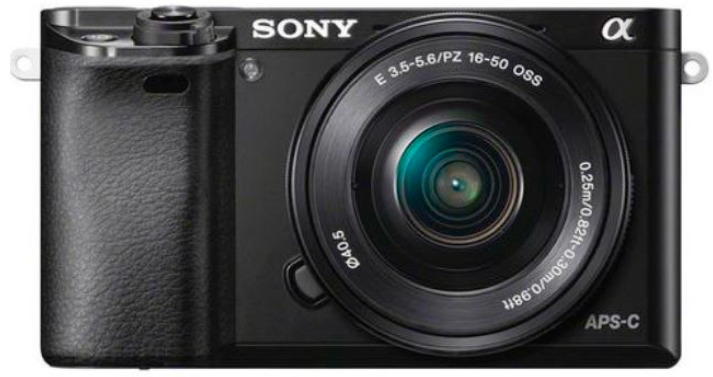
The visible-light camera used in this study.

A visible-light camera ILCE-6000 (Sony Inc., Tokyo, Japan) (Figure 4, Table 3) with an integrated global positioning system (GPS) sensor was attached to the Tuffwing UAV. The visible camera produces 24.3-megapixel-format (6000 × 4000) images in true color bands (red, green, blue) with 8-bit radiometric resolution. These images were stored on a secure digital data card. To achieve the desired forward overlap of 75% between images, the camera was triggered by the UAV’s controller to vary the frame rate based on flight speed.

#### 2.2.3. Flight Control

**Figure 5:**
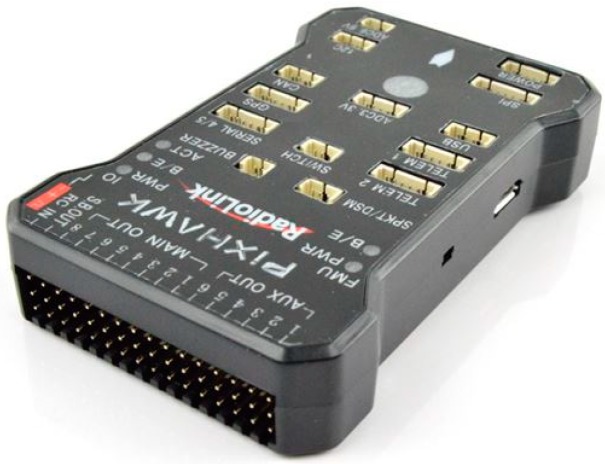
Pixhawk controller for fixed-wing UAV.

The Pixhawk controller (Figure 5, Table 4) used on the UAV includes a computer that autonomously controls flight navigation with the NuttX real-time operating system. Each flight was conducted in the auto-pilot mode with the “Mission Planner” ground station software [37] along flight paths that were based on camera specifications, field area corner coordinates, flying parameters, and an overlap percentage between images.

#### 2.2.4. Flight Procedures

The focal length of the camera lens was a 16 mm with a fixed zoom to achieve a ground resolution of approximately 2.74 cm/pixels at the standard operating procedure with a maximum altitude of 120 m above the ground level (AGL). During flight missions, the UAV was flown at an AGL of 120 m with a ground speed of 17 m/s within 2 h of solar noon. The shutter speed was fixed for each flight with the focus distance set at infinity. During each flight mission, aerial images were captured with an overlap of 75% among both forward and side directions. Images were collected only on cloud-free days.

### 2.3. Ground Control Points (GCPs)

#### 2.3.1. Structure

**Figure 6:**
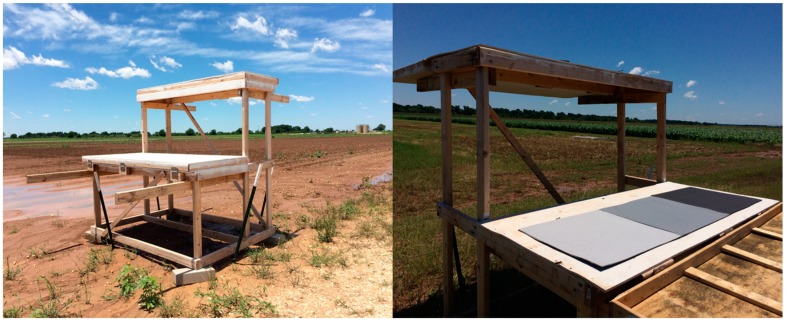
Ground control points set up in the field for geo-referencing, radiometric calibration, and crop height calibration (Photo Credit: Cody Bagnall).

Eight multi-level GCPs (Figure 6) were constructed with wooden frames and affixed to the ground with metal anchors to act as a semi-permanent calibration system, which is located around the field covered by UAV flights. The eight GCPs were used for geo-referencing the field orthomosaic and then five of the eight were also used for plant height calibration only in the sorghum breeding plots. Flights of the entire field required 14 flight paths while the sorghum breeding plots would have required only three flight paths. The DSM of the sorghum breeding plots was clipped out from the DSM of the entire field. Each GCP had two platforms with three 61 cm square radiometric calibration references. Each GCP level is 183 m long with the top level being 61 cm wide and the lower level being 76 cm wide. This gives roughly 500 pixels for each calibration reference, which makes it very easy to precisely identify specific positions on the GCP for purposes of georectification. The radiometric references were used in another study, but in this study, the GCPs were used strictly for geo-referencing and height calibration. The heights from the ground to the bottom and top panels were 91.5 cm and 183 cm, respectively, which took into account the height variation of the sorghum plants from the early vegetative stage to the reproductive stage.

#### 2.3.2. Uses

The GCPs were uniformly distributed across the field (Figure 1a). A Trimble R8 GNSS unit and an R7 base station, accurate to 1 cm + 1 ppm horizontal and 2 cm + 1 ppm vertical after post-correction based on known benchmarks, was used to collect a GPS point at the front left and front right corner of the lower deck for all eight GCPs. Later, the GCPs were identified in the mosaicked images for geo-referencing and height calibration. Because the heights of each GCP level were known, plant height estimates were calibrated based on the GCP platform heights.

### 2.4. Image Data Processing

#### 2.4.1. UAV SfM Method

**Figure 7:**
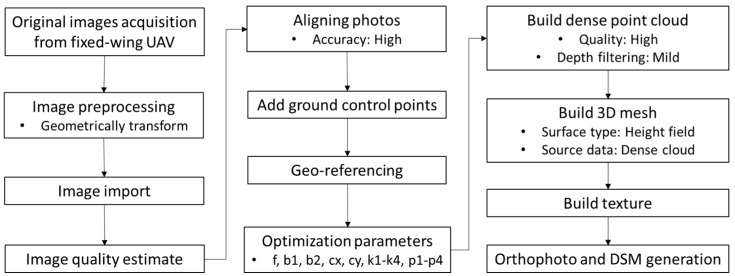
Flowchart of the entire procedure for generating orthomosaic and digital surface model from the collected UAV imagery.

Images collected on each date were mosaicked in PhotoScan Professional 1.3.1 (Agisoft LLC, St. Petersburg, Russia) software and a DSM was calculated with SfM involving interpolation of 3D point clouds, which was accomplished in PhotoScan. The processing steps included aligning images, building a dense point cloud, building a 3D mesh, and building a field geometry (Figure 7, Table 5). In the image-alignment step, the GCP positions were imported, manually located, and matched on images to determine camera position for each image and to refine the camera calibration parameters for the software, which included camera type, focal length, radial distortion coefficients, and tangential distortion coefficients. In the point cloud building step, a dense point cloud model was generated based on the estimated camera positions to provide accurate depth map data for each image overlap area. “Mild” depth filtering—a built-in median filtering algorithm mode—was used to sort the outliers from the generated dense point cloud. In the 3D mesh building step, a 3D polygonal mesh was constructed to produce an estimated crop surface based on the dense point cloud through an algorithm called “Height Field.” In the field geometry building step, the software requires selection of a blending mode for texture generation and this mode was set as the “Mosaic” to generate the orthomosaic and DSM in the *TIF image format.

#### 2.4.2. Crop Height Analysis

**Figure 8:**
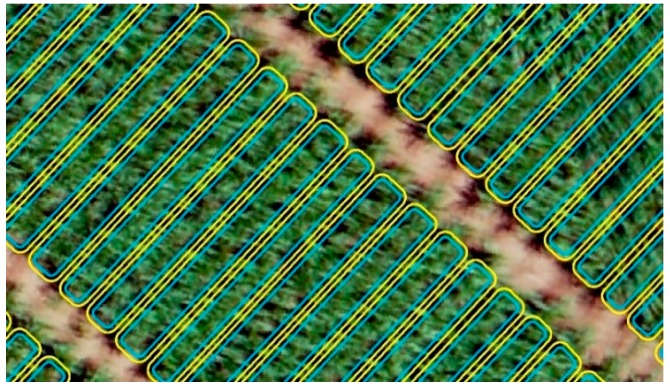
Plot boundaries (yellow lines) and buffered boundaries (blue lines) overlaid on an orthomosaic.

**Figure 9:**
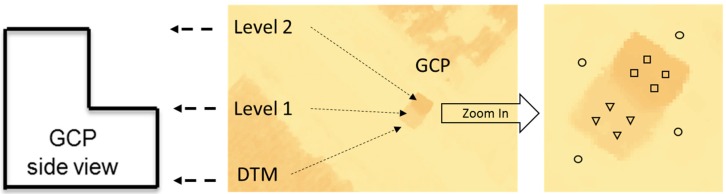
Height calibration using the developed multi-level GCPs in the generated digital surface model (DTM: circles, GCP level 1: triangles, GCP level 2: rectangles).

Plot boundaries were created in ArcGIS 10.3 (ESRI, Redlands, CA, USA) based on the experimental layout dimensions shown in Figure 1. The plot boundaries were then moved inward by 15 cm with the ArcGIS buffer tool in order to exclude edge effects due to the potential foliage encroachment from adjacent plots (Figure 8). The digital terrain model (DTM) was created by using SfM to measure bare ground elevations in the unplanted field and the DSM was created through a combination of the bare ground elevations and the crop features in the field. To perform plant height calibration from the DSM, a linear calibration equation was developed for each flight date (Equation (1)) with three points and were extracted from the DTM, GCP level 1, and GCP level 2 (Figure 9). These three calibration points were the median of four samples from each level at each GCP, ground, lower platform, and upper platform. The samples included four-pixels-sized polygon grids for extracting original height values from the DSM. Height calibration was implemented with aerial measured values of the GCPs based on the derived linear calibration equation. A key step in estimating plant height is subtracting the DTM from the DSM [30,38]. Maximum plant height was extracted as the final plant height for each plot (genotype). Experimental data for UAVB on 7/25 were not included in the analysis since the sample had been harvested earlier.

(1) Calibrated Height=Slope×Original Height±Intercept

where original height is taken from the uncalibrated DSM and calibrated height is taken from the calibrated DSM based on the multi-level GCPs.

#### 2.4.3. Comparison with Ground-Truth Measurements

The estimated plant heights from the fixed-wing UAV were compared to the ground-truth measurements across the 700 sorghum plots that uniformly distributed with fixed gaps (0.76 m) between the plots. Coefficient of determination (R^2^), root mean square error (RMSE), and relative RMSE as shown in Equation (2) were calculated for each date and genotype. Trends relative to over or underestimation in the ground-truth and UAV-estimated data were considered. Moreover, the improvements in accuracy with GCP-based calibration were also considered.

(2) $$\mathrm{Relative}\;\mathrm{RMSE}=100\%\times \frac{\sqrt{\frac{1}{n}\sum_{i=1}^{n}{\left(x_{i}-{\hat{x}}_{i}\right)}^{2}}}{\frac{1}{n}\sum_{i=1}^{n}x_{i}}$$

where n is the number of plots, $x_{i}$ is the ground-truth plant height for plot i, and ${\hat{x}}_{i}$ is the UAV-estimated plant height for plot i.

### 2.5. Image Quality Assessment

**Figure 10:**
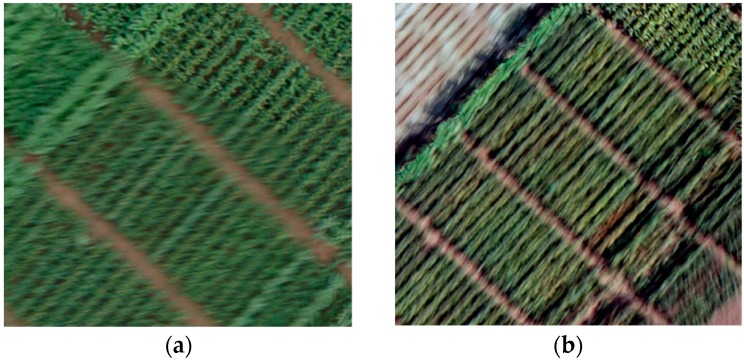
Blurred images captured on (**a**) June 16 and (**b**) July 25.

**Figure 11:**
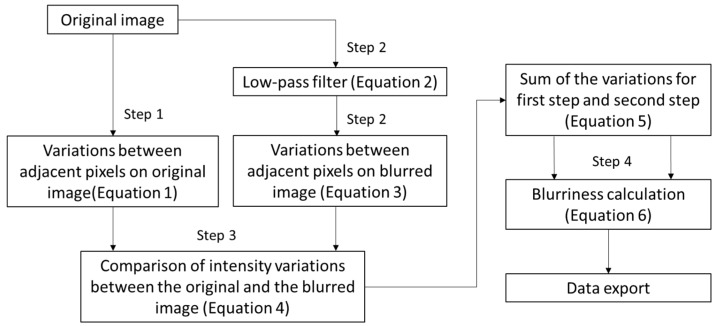
Flowchart of processing of the image quality assessment (Crete et al., 2007).

Image blur can reduce DSM accuracy [39] due to camera vibration, and image-object motion, during flight (Figure 10). Thus, image quality assessment was performed with a method called “no-reference blur estimation” to quantify the blurriness of images. This method was discussed by Crete et al. [40] and it discriminates between different levels of blur perceptible in the same base image (Figure 11).

In the first step of calculating blurriness, the intensity variations between adjacent pixels of the original mosaicked image were calculated. Equation (3) involves the absolute values of the variations between adjacent pixels in an original m × n image’s horizontal (Δp1) and vertical (Δp2) directions.

(3) $$\Delta p1=\left|p1_{j}^{i}-p1_{j}^{i-1}\right|,\;\Delta p2=\left|p2_{j}^{i}-p2_{j-1}^{i}\right|$$

In the second step, a low-pass filter (Equation (4)) was used on the original mosaicked image to reduce the variations between the adjacent pixels. Equation (5) involves the variation of adjacent pixels in the horizontal (Δq1) and vertical (Δq2) directions of the blurred image.

(4) $$h1=\frac{1}{4}\left[111111111\right],\;h2=h1^{\prime }$$

(5) $$\Delta q1=p1\times h1=\left|q1_{j}^{i}-q1_{j}^{i-1}\right|,\;\Delta q2=p2\times h2=\left|q2_{j}^{i}-q2_{j-1}^{i}\right|$$

In the third step, the image blurriness was evaluated through a comparison of intensity variations between the original image and the blurred image. Equation (6) involves the variation of adjacent pixels between the original and blurred images in the horizontal (Δu1) and vertical (Δu2) directions. A high variation indicates that the original image was clear. Otherwise, the original image was already somewhat blurred.

(6) Δu1=max(0, Δp1−Δq1), Δu2=max(0, Δp2−Δq2)

Lastly, Equation (7) involves the sum of the calculated intensity variations for the second and third steps. The evaluated image blurriness was normalized in a defined range from 0 to 1. In addition, the final blurriness of the image was defined in the horizontal (η1) and vertical (η2) directions, which is shown in Equation (8).

(7) $$\eta 1=1-\frac{\sum_{i=1,j=1}^{m-1,n-1}\Delta u1}{\sum_{i=1,j=1}^{m-1,n-1}\Delta p1},\;\eta 2=1-\frac{\sum_{i=1,j=1}^{m-1,n-1}\Delta u2}{\sum_{i=1,j=1}^{m-1,n-1}\Delta p2}$$

(8) blurriness=max(η1, η2)

The effects of image quality on plant height measurement were observed by considering the relationship between image blur and plant-height error and image blurriness was compared to wind speed measured with a nearby ground-based weather station on each flight date to examine the cause of the image blur.

## 3. Results

### 3.1. Plant Height Estimation with Fixed-Wing UAV

#### 3.1.1. SfM Model Accuracy and Trends in Ground-Truth and Estimated Plant-Height Data

The RMSEs of the GCP coordinates over five flights are shown in Table 6. The X-coordinate RMSEs ranged from 1.83 to 2.52 cm, the Y-coordinate RMSEs ranged from 1.72 to 3.09 cm, and the Z-coordinate RMSEs ranged from 0.96 to 2.22 cm, which indicates that the geo-referencing data provided positioning accuracy well under 4 cm in all coordinate directions.

**Figure 12:**
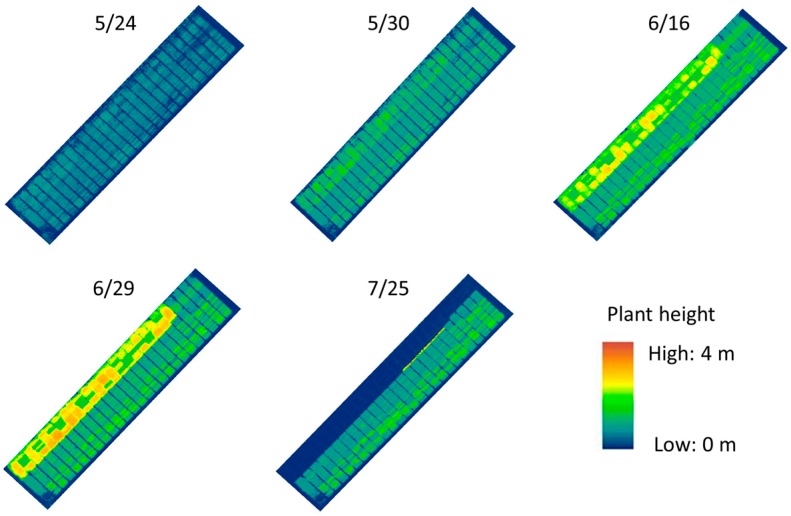
Height comparison datasets with the digital surface model on 05/24, 05/30, 06/16, 6/29, and 07/25 in the sorghum field.

**Figure 13:**
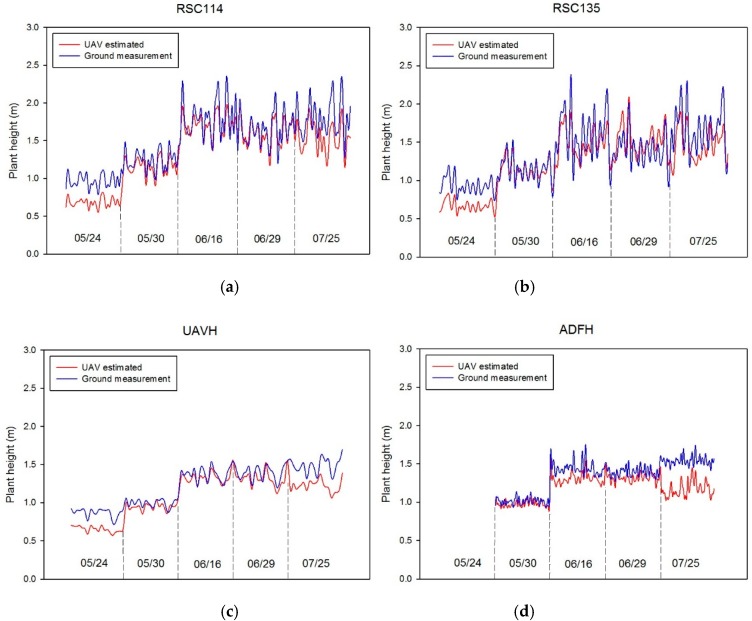

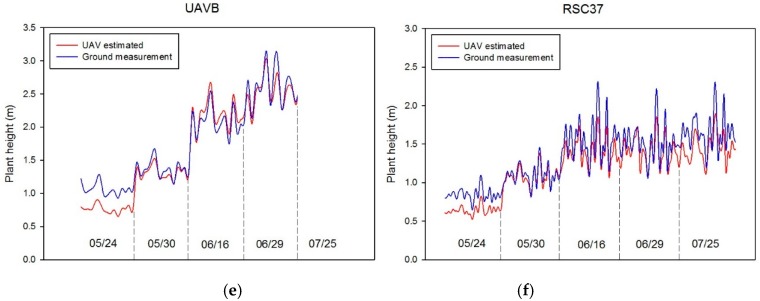
Changes in crop height trends for sorghum at the six experimental tests on (**a**) RSC114, (**b**) RSC135, (**c**) UAVH, (**d**) ADFH, (**e**) UAVB, and (**f**) RSC37 over five flights.

Figure 12 shows the digital surface model outputs of the test sorghum field. Changes in crop coverage and plant height were well represented in terms of plant growth across the five dates. As mentioned previously, experimental data for UAVB on 7/25 were not included in the analysis. Figure 13 shows a comparison between UAV-estimated plant height and the standard ground-based method. Plant height measurements varied significantly at different growth stages across the five dates from May to July 2017. Estimates from UAV-based and field measurements showed consistent crop growth up to June 16 and a levelling off afterward (except for UAVB). Measurement biases were evident on May 24. Plant height errors were in the range of 0.161 to 0.222 m for both experimental tests during the plants’ early vegetative stage. Furthermore, UAV estimates on July 25 also appeared to underestimate plant height during the plants’ reproductive stage. Errors ranged from 0.197 to 0.320 m.

#### 3.1.2. Accuracy Assessment of SfM Plant-Height Estimates

**Figure 14:**
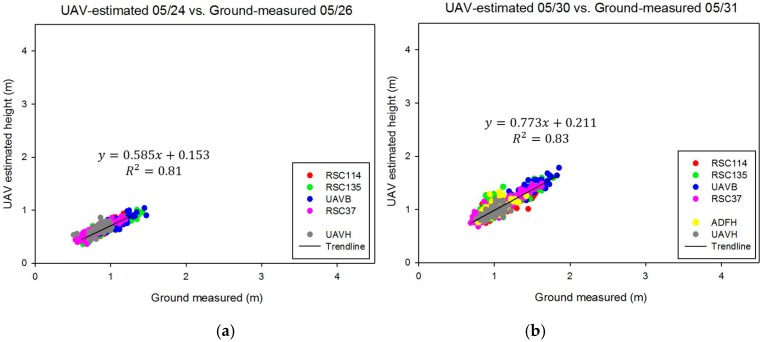

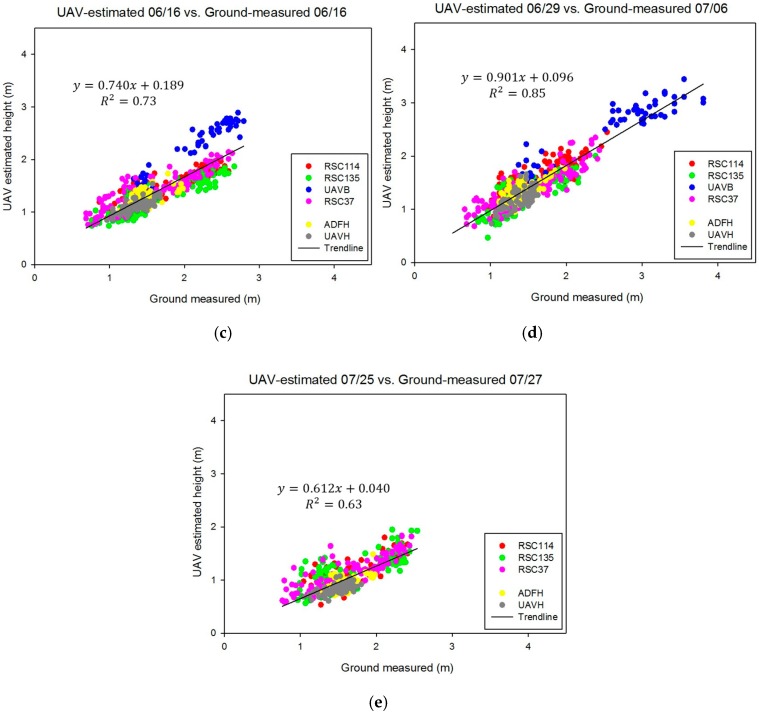
Correlations between UAV-estimated plant height and ground-truth plant height for all experimental tests over five flights on (**a**) 05/24, (**b**) 05/30, (**c**) 06/16, (**d**) 06/29, and (**e**) 07/25.

**Figure 15:**
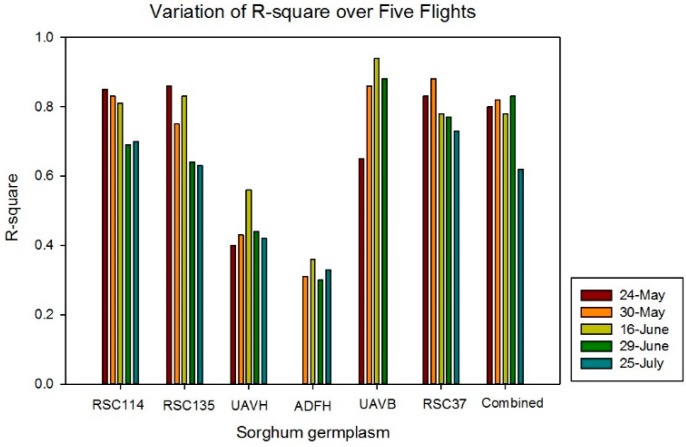
Variation of R^2^ for sorghum height estimate over five flights.

**Figure 16:**
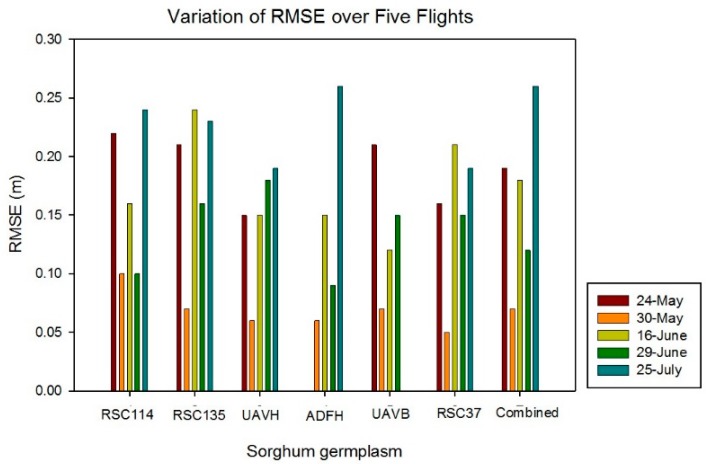
Variation of RMSE for sorghum height estimate over five flights.

Strong correlations between UAV estimates and ground-truth measurements were determined (R^2^ = 0.80, 0.82, 0.72, 0.88, and 0.62 for May 24, May 30, June 16, June 29, and July 25, respectively), which is shown in Figure 14. This implies that SfM is effective in estimating the heights of sorghum. The UAV data estimated on June 16 did not fit with the ideal line well because of the effects of the image blurring. Strong linear relationships (R^2^ > 0.70) generally existed between UAV estimates and ground-truth measurements for most of the sorghum germplasm types, which is shown in Figure 15. However, since expected correlations were weaker for the advanced hybrids UAVH and ADFH, which had relatively low height variation because of the lesser range in the height data (yellow and gray dots in Figure 14). When all experimental tests were combined into one data set, the R^2^ value was around 0.80 for each flight date except July 25 (R^2^ = 0.62). The RMSE comparison between UAV estimates and ground-truth measurements for all sorghum germplasm types is shown in Figure 16. Relatively low RMSE values (<0.20 m) existed for May 30, June 16, and June 29. Furthermore, the RMSE values for each flight date on all the combined experimental tests were lower than 0.2 m except for July 25 (0.26 m).

### 3.2. Plant Height Accuracy Improvement with Height Calibration

**Figure 17:**
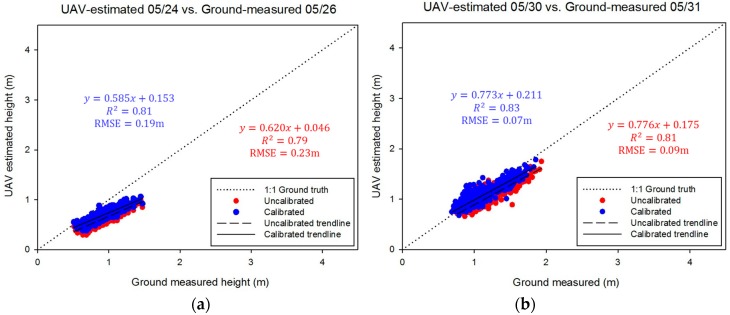

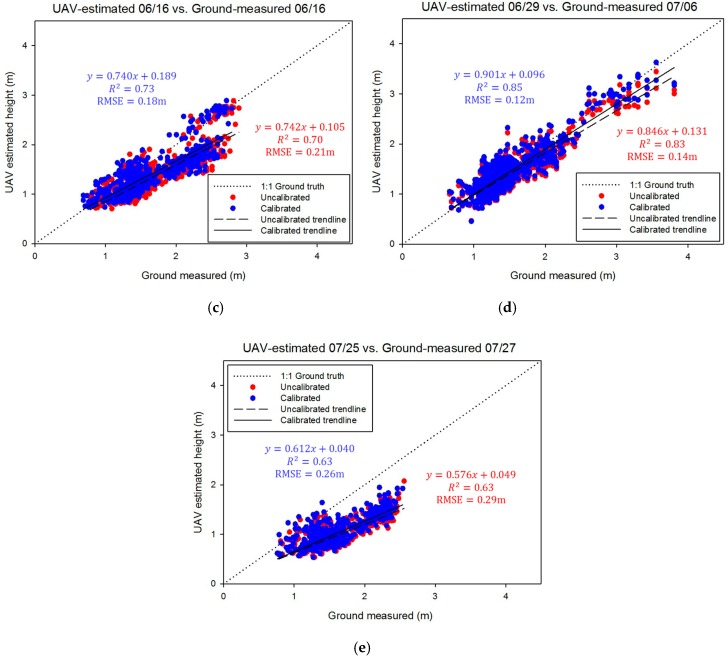
Comparison of uncalibrated data and calibrated data through the height calibration method over five flights on (**a**) 05/24, (**b**) 05/30, (**c**) 06/16, (**d**) 06/29, and (**e**) 07/25. The black dotted line indicates a 1:1 ground truth line.

Linear calibrations of UAV-based plant height estimates did not significantly increase the correlations between UAV estimates and ground truth (Figure 17). However, calibration did significantly reduce the RMSE values. The trend lines of the calibrated data were closer to the 1:1 ground truth line than the uncalibrated trend line on every date due to the biases present in the original DSM (uncalibrated data) in which the actual value of the ground surface and actual maximum plant height were unknown. Therefore, the calibration appears to reduce the inherent bias in plant height data. The relative RMSEs in relation to measured heights for each flight date are given in Table 7, which shows an overall downward trend during the season, as might be expected because the error is relatively consistent when the plants get taller. Furthermore, the calibration reduced RMSE to 0.19 m, 0.07 m, 0.18 m, 0.12 m, and 0.26 m for May 24, May 30, June 16, June 29, and July 25, respectively. Thus, the error in plant height estimates was reduced by around 20% (21%, 29%, 18%, 17%, and 13% for May 24, May 30, June 16, June 29, and July 25, respectively) overall with calibration.

### 3.3. Plant Height Accuracy Correlation with Image Quality

**Figure 18:**
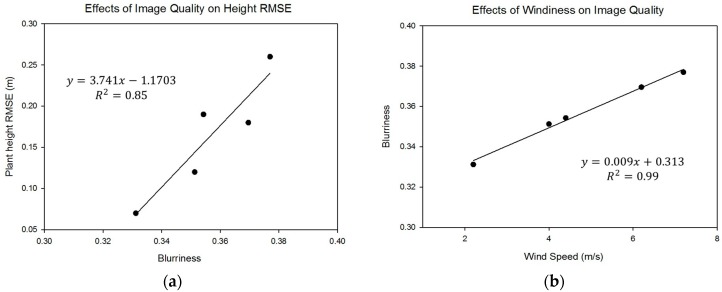
(**a**) Effects of image quality on height RMSE and (**b**) effects of windiness on image quality.

As exemplified in Figure 10, some images were blurry, which makes it difficult in those cases to obtain high-accuracy plant height estimates. This problem was particularly acute on July 25 when the RMSE was at its highest at 0.26 m. As previously mentioned, the level of blur in each mosaicked image was quantified and a strong correlation (R^2^ = 0.85) was observed between image quality and plant height RMSE, which is shown in Figure 18a. Low blurriness was associated with low plant height RMSE. The influence of wind speed was considered and a strong linear relationship (R^2^ = 0.99) was found to exist between wind speed on the time of flight and image blurriness, which is shown in Figure 18b. Thus, it is strongly suggested that weather conditions play a significant role in the accuracy of plant height estimates from SfM with images collected by a fixed-wing UAV at 120 m AGL.

**Figure 19:**
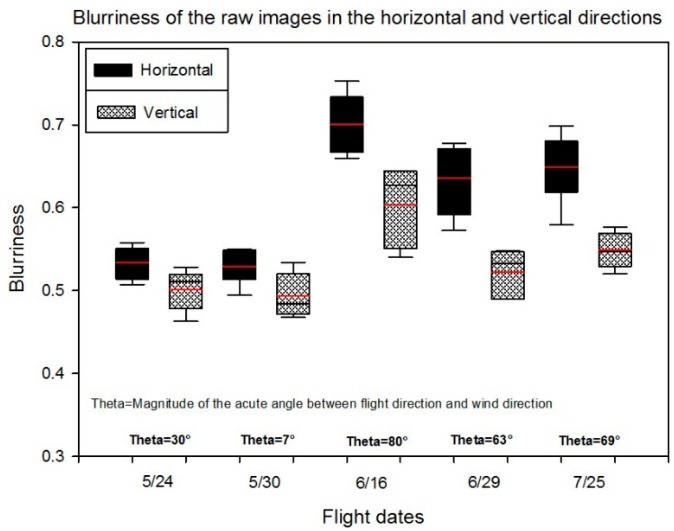
Blurriness of the raw images in the horizontal and vertical directions.

Fixed-wing aircraft commonly use banking turns to maintain the flight-path direction when crosswinds occur. If the aircraft rolls back and forth slightly in response to an unsteady crosswind, the viewing angle toward the ground can change significantly very quickly, which may potentially cause the image to blur. Therefore, it was expected that wind would have a stronger effect on the blurriness of UAV images if the wind were across the flight path rather than along the flight path. The effects of crosswinds were expected to be more severe in the horizontal direction in the images than in the vertical direction. To evaluate this idea, the blurriness of raw images over five flights was assessed in the horizontal and vertical directions, which is shown in Figure 19. The blurriness in the horizontal direction was greater than in the vertical direction for each flight and the blurriness in the horizontal direction was much greater than in the vertical direction on the windier days of 6/16 and 7/25. Furthermore, there was more blurriness if the wind direction was across the UAV flight path. More blurriness and a greater difference between blurriness in the horizontal and vertical directions occurred on the last three flight days (6/16, 6/29, and 7/25), which had larger acute angles between flight paths and wind directions (80°, 63°, and 69°).

## 4. Discussion

As mentioned previously, Malambo et al. used SfM to estimate sorghum plant height from rotary-wing UAV imagery [30]. Strong linear relationships between UAV-based and manually measured plant height for sorghum (R^2^ = 0.67–0.85) with relatively low RMSE values (0.12–0.24 m) were observed. Furthermore, Chang et al. proposed a framework for sorghum plant height monitoring with UAS data and identifying that the RMSE between field measurements and the proposed approach was 0.33 m based on rotary-wing UAV [38]. These results are consistent with other studies [14,19] focused on other crops. From a production-agriculture point of view, it is important to consider whether fixed-wing aircraft flying higher that typical rotary-wing flights can produce accurate plant-height estimates. In addition, being able to measure plant height over large areas could increase the speed of plant breeding programs by increasing the number of plots monitored. Thus, this study involved estimating sorghum plant height with images from a fixed-wing UAV at 120 m AGL. The plant-height estimates showed a strong linear relationship (R^2^ > 0.70) with ground-truth measurements and RMSE values (RMSE < 0.20 m) were generally small and comparable to those of previous studies with rotary-wing UAVs at lower AGLs. Therefore, it is clear that fixed-wing UAVs at 120 m AGL have the potential to estimate plant height, which enables measurements to be made over relatively larger fields that could not be covered with standard hand based methods and might be too large for rotary-wing UAVs. Some varieties in this study especially the early generation hybrids in the pollinator inbred line had relatively high height variation and, thus, relationships were stronger because of the greater range in the height data. In contrast, the advanced hybrids had relatively low height variation and, thus, relationships were weaker because of the lesser range in the height data.

While it is evident that fixed-wing UAVs at 120 m AGL have the potential to estimate plant height, it is critical to develop methods that are accurate and provide repeatable data. Proposed methods to reduce error in plant-height measurements have been lacking in the literature. This study considered two types of error: (1) height biases due to errors in the DTM and DSM and (2) the effects of wind. The method proposed to reduce plant height biases involved calibration of the DSM based on multi-level GCPs in the field at the time of flight. Results indicated that height calibration was capable of significantly improving plant-height estimates (RMSE improvement ≈ 20%). This reduction in error has important implications. Decisions regarding irrigation, fertilizer, and more are often based on projected crop yield and, in many crops, there is a strong relationship between yield and plant height [41]. Therefore, more accurate height measurements should result in improved on-farm decision-making.

In addition to errors in the image-based DTM and DSM, it is important to consider possible errors in the ground-truth data against which the UAV data are compared. For example, human data collectors have a downward-looking perspective for height measurements during the plant’s early vegetative stage. While the UAV measurements appeared to underestimate height on May 24, it is likely that human error contributed to these inaccuracies. In addition, there was concern about errors resulting from poor formation of point clouds that could result from the effects of wind either on the aircraft or from the plants in the field. It is notable that plant height RMSE was strongly related to image blurriness and image blurriness was strongly related to wind speed on the day of flight (Figure 18). Furthermore, the influence of wind speed and direction on image blurriness in the horizontal and vertical directions was analyzed to show the relationship between wind effects and image quality. It is apparent that crosswinds had strong effects on plant height estimates. Thus, it is suggested that weather conditions can play a major role in the accuracy of plant height estimates from fixed-wing UAV images collected at 120 m AGL [19,42]. Further research needs to be done to improve 3D point cloud accuracy by understanding and overcoming the sources of blurriness in captured images and plant movement between overlapping images.

It is notable that UAV measurements also underestimated plant height on July 25. This effect may have been caused by image blur or plant movements resulting from high wind speeds. As can be seen in Table 1, the wind speed measured by a nearby weather station was 7.2 m/s on July 25, which is the highest value over five flights. Previous studies [19,42] have indicated problems related to creating high-resolution maps in windy conditions or when various noise effects in point clouds exist [43,44]. Overall, results of this study indicate that fixed-wing UAV images collected at 120 m AGL can be used to estimate sorghum plant height and growth trends reasonably well and multi-level GCPs are helpful in reducing error on relatively flat terrain. However, some error sources like weather conditions remain problematic. Some prior studies [45,46] have focused on conventional GCP applications in complex topography. It should be possible to effectively apply height calibration with multi-level GCPs over rough terrain if the GCPs are appropriately distributed across the field.

## 5. Conclusions

This work indicates the feasibility of using SfM on images collected from 120 m AGL with a fixed-wing UAV to estimate sorghum plant height with reasonable accuracy on a relatively large farm field. UAV-based plant height estimates on multiple dates were able to highlight trends in plant growth. Discrepancies between UAV-based estimates and ground truth existed during the vegetative stage, but this difference is likely caused by inaccuracy of ground truth due to the human viewing perspective. Correlations between UAV-based estimates and ground truth were strong on all dates but were clearly better on some dates than others. Furthermore, a new method for improving UAV-based plant height estimates with multi-level GCPs was found to lower RMSE by about 20%. These results indicate that multi-level GCP-based height calibration has a potential for future application where accuracy is particularly important. Lastly, the image blur appeared to have a significant impact on the accuracy of plant height estimation. A strong relationship (R^2^ = 0.85) was observed between image quality and plant height RMSE and the influence of wind was a challenge in obtaining high-quality plant height data. A strong linear relationship (R^2^ = 0.99) was identified between wind speed and image blurriness. Image blur can also be caused by improper camera settings and care must be taken to ensure that camera shutter speed is fast enough for fixed-wing UAV flights. In the future, different lenses or exposure times should be investigated to reduce the error of the plant height estimation.

## Figures and Tables

**Table 1:** UAV flights with corresponding field plant height measurements.

Flight Date	Ground Truth Date	Days Difference	Number of Images	Number of Plots	Wind Speed
05/24	05/26	2	231	700	4.4 m/s
05/30	05/31	1	242	700	2.2 m/s
06/16	06/16	0	242	700	6.2 m/s
06/29	07/03	4	233	700	4.0 m/s
07/25	07/27	2	240	610	7.2 m/s

**Table 2:** Specifications of the fixed-wing unmanned aerial vehicle (UAV) platform.

Items	Specifications
Wingspan	1.22 m
Weight maximum	2 kg
Material	EPP foam, carbon fiber tubes, coroplast
Battery	6200 mAh, lithium polymer
Flight planning software	Mission Planner
Endurance	40 minutes
Minimum air speed	16 meters per second

**Table 3:** Specifications of the visible-light camera.

Items	Descriptions	Specifications
Sensor	Sensor	APS-C type (23.5 × 15.6 mm)
	Number of pixels	24.3 MP
	Image sensor aspect ratio	3:2
Exposure	ISO sensitivity	ISO 100-25600
Shutter	Shutter speed	1/4000 to 30 s
	Flash sync. speed	1/160 s
Lens	Focal length Aperture range	16 mm F22 to F2.8
Size and Weight	Dimensions (W × H × L)	4.72 × 2.63 × 1.78 in
	Weight (with battery)	0.34 kg

**Table 4:** Specifications of the Pixhawk controller for the fixed-wing UAV.

Items	Specifications
Processor	32-bit ARM Cortex M4 core with FPU
	168 Mhz/256 KB RAM/2 MB Flash
	32-bit failsafe co-processor
Sensors	MPU6000 as main accel and gyro
	ST Micro 14-bit accelerometer/compass (magnetometer)
	ST Micro 16-bit gyroscope
Dimensions (W × H × L)	2.0 × 0.6 × 3.2 in
Weight	3.8 g

**Table 5:** Parameters of the UAV flights and Agisoft Photoscan processing used in the study.

Items	Descriptions	Values
Alignment	Accuracy	High
	Adaptive camera model fitting	Yes
Dense point cloud	Quality	High
	Depth filtering	Mild
DEM	Model resolution	Around 5.52 cm/pix
	Source data	Dense cloud
Orthomosaic	Coordinate system	WGS 84/UTM zone 14N
	Blending mode	Mosaic

**Table 6:** RMSE at GCP locations for the SfM model over five flights.

Flight Date	X_RMSE (cm)	Y_RMSE (cm)	Z_RMSE (cm)
5/24	2.52	1.72	1.88
5/30	2.23	2.12	0.96
6/16	2.29	1.96	1.83
6/29	1.83	3.09	2.22
7/25	1.87	2.55	2.18

**Table 7:** Accuracy improvement results between uncalibrated data and calibrated data from the height calibration method over five flights.

Date	Performance				
	Uncalibrated RMSE	Calibrated RMSE	Improvement RMSE	R2	Relative RMSE
05/24	0.23 m	0.19 m	21.3%	0.81	20.4%
05/30	0.09 m	0.07 m	29.2%	0.83	6.1%
06/16	0.21 m	0.18 m	17.7%	0.73	12.0%
06/29	0.14 m	0.12 m	17.4%	0.85	8.0%
07/25	0.29 m	0.26 m	12.8%	0.63	16.2%
